# Non-Magnetic Bimetallic MOF-Derived Porous Carbon-Wrapped TiO_2_/ZrTiO_4_ Composites for Efficient Electromagnetic Wave Absorption

**DOI:** 10.1007/s40820-021-00606-6

**Published:** 2021-02-17

**Authors:** Jing Qiao, Xue Zhang, Chang Liu, Longfei Lyu, Yunfei Yang, Zhou Wang, Lili Wu, Wei Liu, Fenglong Wang, Jiurong Liu

**Affiliations:** 1 School of Materials Science and Engineering, Shandong University, Jinan, 250061 People’s Republic of China; 2 State Key Laboratory of Crystal Materials, Shandong University, Jinan, 250100 People’s Republic of China

**Keywords:** Bimetallic metal–organic framework, PCN-415, MOF derivatives, TiO_2_/ZrTiO_4_/C composites, Electromagnetic wave absorption

## Abstract

**Highlights:**

- Non-magnetic bimetallic MOF-derived porous carbon-wrapped TiO_2_/ZrTiO_4_ composites are firstly used for efficient electromagnetic wave absorption.
- The electromagnetic wave absorption mechanisms including enhanced interfacial polarization and essential conductivity are intensively discussed.

**Abstract:**

Modern communication technologies put forward higher requirements for electromagnetic wave (EMW) absorption materials. Metal–organic framework (MOF) derivatives have been widely concerned with its diverse advantages. To break the mindset of magnetic-derivative design, and make up the shortage of monometallic non-magnetic derivatives, we first try non-magnetic bimetallic MOFs derivatives to achieve efficient EMW absorption. The porous carbon-wrapped TiO_2_/ZrTiO_4_ composites derived from PCN-415 (TiZr-MOFs) are qualified with a minimum reflection loss of − 67.8 dB (2.16 mm, 13.0 GHz), and a maximum effective absorption bandwidth of 5.9 GHz (2.70 mm). Through in-depth discussions, the synergy of enhanced interfacial polarization and other attenuation mechanisms in the composites is revealed. Therefore, this work confirms the huge potentials of non-magnetic bimetallic MOFs derivatives in EMW absorption applications.
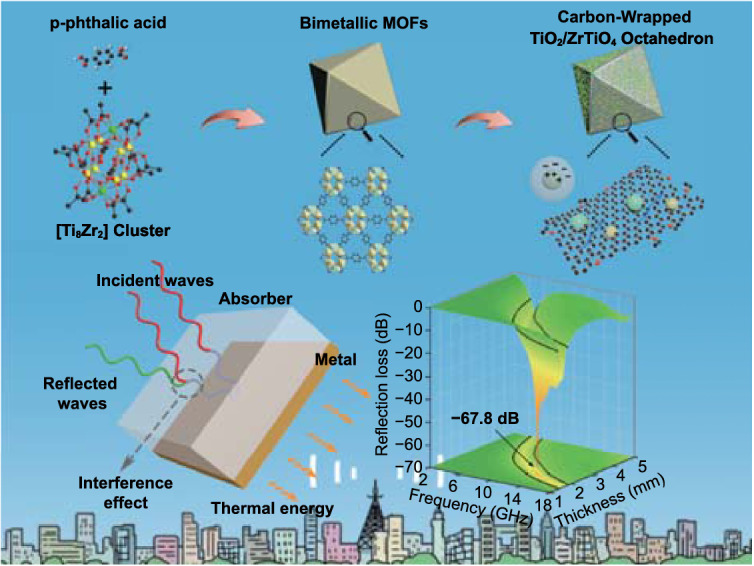

**Supplementary information:**

The online version contains supplementary material available at (10.1007/s40820-021-00606-6).

## Introduction

The deterioration of electromagnetic environment caused by modern communication technologies has put forward higher requirements for electromagnetic wave (EMW) absorption materials in physical protection and electronic equipment anti-interference [1–4]. Among various selectable materials, the carbon-based metal–organic framework (MOF) derivatives have been gradually considered as a significative category for efficient EMW absorption because of the superiorities in porosity, uniformity, alterable composition, controllable microstructure, and so on [5–7]. In addition, the flexible preparation and mild conditions in synthetic process qualify them with huge potentials in practical production. In the nascent stages, the researchers have already confirmed their performance advantages in EMW absorption. For example, Wang et al. [8] prepared porous Co–C core–shell nanocomposites derived from Co-MOF-74, achieving the synergy of magnetic and dielectric loss to enhance the absorption intensity (− 62.12 dB). Wu et al. [9] testified the improved impedance matching condition in Fe-containing magnetic nanoparticles embedded carbon rods by carbonizing the Fe-MIL-88A, and a thin matching thickness (− 45.4 dB, 1.58 mm) was obtained. Liao et al. [6] synthesized Co/ZnO/C microrods derived from bimetallic CoZn-MOFs. Making use of the enhanced interface polarization, the effective absorption bandwidth (EAB) was broadened evidently (4.9 GHz, 3.0 mm). As these examples indicated, most researchers focused on the investigation of magnetic MOF derivatives because high permeability arising from magnetic components would optimize the impedance matching characteristics, which is beneficial to strengthen the absorption intensity and reduce the matching thickness [10–13]. However, the magnetic nanoparticles such as Fe, Co, Ni, and their alloys are suffering from high material density and low chemical stability [14], which limited their further practical application.

Exactly for above reasons, non-magnetic MOF derivatives ignored previously were revived. In recent time, some sporadic reports about non-magnetic composites derived from monometallic MOFs emerged, such as the ZnO/N-doped porous carbon derived from ZIF-8 [15], and the octahedral ZrO_2_ embedded carbon derived from UIO-66 [16]. These studies proved the non-magnetic compositions in carbon base would also regulate the impedance matching characteristic, and epitomized a promise as EMW absorption materials. However, the restrictions such as high filling rate and low absorption intensity still hinder the wide applications of MOF derivatives for efficient microwave absorption. Thus, to prepare double-metallic-oxide composites derived from bimetallic MOFs is worth while trying. Through this strategy, more species of metal oxide/carbon interfaces could further enhance the interfacial polarization to strengthen the attenuation capacity [17, 18]. Combined with the essential conductivity property, strong EMW attenuation characteristics could be achieved. Therefore, the obtained MOF derivatives would not only inherit the superiority in density and stability, but also overcome the weakness in filling rate and absorption intensity.

TiO_2_ and ZrTiO_4_ as microwave dielectric ceramic materials exhibited huge application potentials due to the huge abundance, environmental friendliness, and corrosion resistance. Meanwhile, different from other metallic ions, Ti^4+^ and Zr^4+^ qualified superior high-temperature stability, which ensured that they would form stable oxides rather than be reduced into metallic state in the direct carbonization. This advantage could notably simplify the synthetic process. Therefore, the bimetallic PCN-415 (TiZr-MOFs) was chosen.

In this work, we first applied non-magnetic bimetallic MOF derivatives to achieve efficient EMW absorption. The porous carbon-wrapped TiO_2_/ZrTiO_4_ composites derived from PCN-415 (TiZr-MOFs) exhibited a minimum reflection loss (*RL*) value of − 67.8 dB (2.16 mm, 13.0 GHz), and a maximum effective absorption bandwidth (EAB) of 5.9 GHz (2.70 mm). The performance advantages were originated from the synergy of structures and functions, which ensured the optimized impedance matching, rational conductive loss, and enhanced interfacial polarization. This work confirmed the EMW absorption potential of non-magnetic bimetallic MOF derivatives and manifested the superiorities of the TiO_2_/ZrTiO_4_/carbon composites in practical EMW absorption applications.

## Experimental Section

### Synthesis of MIL-125-Derived TiO_2_/C Nanocomposites (TC-7)

Firstly, 3.0 g of p-phthalic acid (C_8_H_6_O_4_) was dissolved in the mixture of 6 mL of methyl alcohol (MeOH) and 54 mL of N,N-dimethylmethanamide (DMF) to obtain a homogeneous solution. Subsequently, 1.56 mL of titanium(IV) isopropoxide (Ti(OiPr)_4_) was dissolved in the solution with continued stirring for several minutes. Then, the solution was transferred into a Teflon-lined stainless-steel autoclave to heat at 150 °C for 24 h. The MIL-125 was collected after centrifugation, washing by MeOH, and drying at 60 °C. Finally, the MIL-125 powders were carbonized at 700 °C under N_2_ atmosphere for 2 h.

### Synthesis of UIO-66-Derived ZrO_2_/C Nanocomposites (ZC-7)

Firstly, 8 mmol of ZrCl_4_ was dispersed into the mixture of 200 mL of DMF and 110 mL of acetic acid (HAc), and 8 mmol of p-phthalic acid was dissolved in 200 mL of DMF, respectively. Subsequently, the two kinds of solutions were mixed with vigorous stirring. Then, the obtained solution was separated by several glass vials and heated at 120 °C for 24 h. The UIO-66 was collected after centrifugation, washing by DMF, and drying at 60 °C. Finally, the UIO-66 powders were carbonized at 700 °C under N_2_ atmosphere for 2 h.

### Synthesis of PCN-415-Derived TiO_2_/ZrTiO_4_/C Nanocomposites (TZC)

0.5 g of ZrCl_4_ was dispersed in the mixture of 50 mL of DMF and 5 mL of HAc, and subsequently, 1 mL of Ti(OiPr)_4_ was dissolved in the solution with stirring for several minutes. Then, the obtained solution was separated by several glass vials and heated at 100 °C for 24 h to obtain the Ti-Zr cluster solution.

4 g of p-phthalic acid was dissolved in the mixture of 50 mL of DMF and 5 mL of trifluoroacetic acid (CF_3_COOH). Subsequently, the whole Ti-Zr cluster solution was poured inside, and the final mixture was separated by several glass vials to heat at 140 °C for 24 h.

The PCN-415 was collected after centrifugation, washing by DMF, and drying at 60 °C. Then, the obtained precursor was carbonized under N_2_ atmosphere for 2 h at 600, 700, 800, and 900 °C, respectively. The obtained black powder samples were marked as TZC-6, TZC-7, TZC-8, and TZC-9.

### Synthesis of Toroidal Paraffin Composite Samples

The abovementioned powder samples were, respectively, mixed uniformly with melted paraffin wax at the mass ratio of 35:65, and then shaped into annulus (*Ф*_in_, 3.04 mm; *Ф*_out_, 7.00 mm) at room temperature by a special mold to measure the electromagnetic parameters.

### Characterizations

The crystalline structure was characterized by powder X-ray diffraction (PXRD; DMAX-2500PC). The micromorphology was obtained by using the field-emission scanning electron microscopy (FE-SEM; Hitachi Model SU-70) coupled with an energy-dispersive X-ray spectroscopy (EDS; X-max), and the high-resolution transmission electron microscopy (HR-TEM; JEM-F200). The contents of carbon were evaluated by thermogravimetric analysis (TGA; HCT-1). The Raman spectra were obtained through a Raman spectrometer (Horiba LabRAM HR). N_2_ absorption–desorption isotherms were recorded by a chemisorption analyzer (Quantachrome Autosorb IQ). The specific surface area and pore-size distribution were calculated by the Brunauer–Emmett–Teller model and Barrett–Joyner–Halenda method, respectively. The surface electronic properties were investigated by X-ray photoelectron spectroscopy (XPS; Thermo ESCALAB 250XI). The Fourier transform infrared (FT-IR) spectra were recorded by a FT-IR spectrometer (VERTEX-70). The conductive properties were recorded by Hall Effect Measurement System (Ecopia HMS-5000). The electromagnetic parameters in the 2.0 − 18.0 GHz were measured by a vector network analyzer (VNA; Agilent PNA N5244A).

## Results and Discussion

### Composition and Structure

**Fig. 1:**
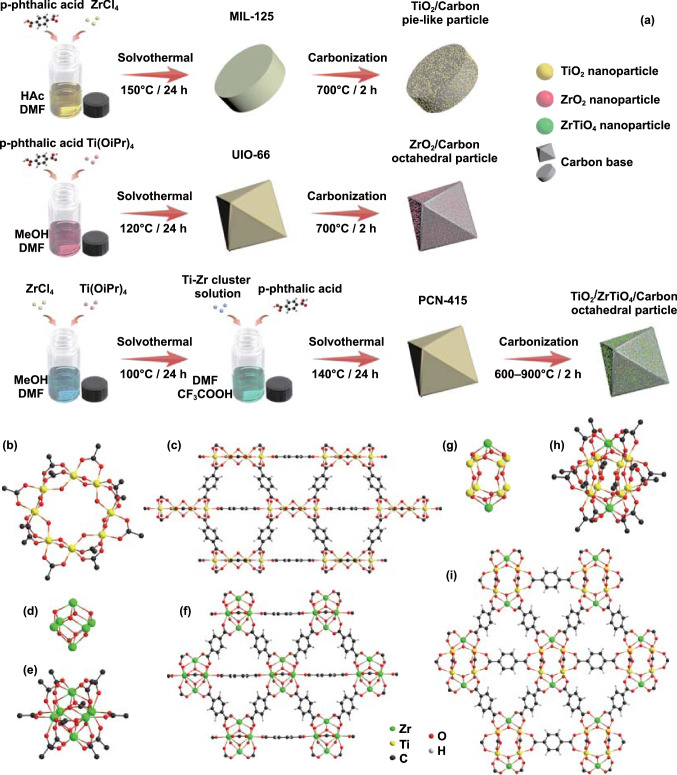
Schematic illustrations for **a** synthesis of MIL-125, UIO-66 and PCN-125 derivatives, **b** Ti-oxo cluster in MIL-125. **c** Crystal structure of MIL-125, **d** Zr_6_ core in UIO-66, **e** Zr-oxo cluster in UIO-66. **f** Crystal structure of UIO-66, **g** Ti_8_Zr_2_ core in PCN-415, **h** TiZr-oxo cluster in PCN-415. **i** Crystal structure of PCN-415

The schematic illustration for synthesis processes of carbon-based MIL-125, UIO-66, and PCN-125 derivatives was shown in Fig. 1a, which mainly including the solvothermal reaction and carbonization. Though different metallic clusters were applied, all MOFs utilized the same organic ligands, *p*-phthalic acid as the frameworks to ensure similar compositions. The internal structures of different MOFs were further discussed, which were illuminated in Fig. 1b–i. In terms of metal oxide clusters, the TiZr-oxo clusters ([Ti_8_Zr_2_O_12_(COO)_16_]) in PCN-415 were more similar to the Zr-oxo clusters ([Zr_6_O_8_(COO)_12_]) in UIO-66, rather than the plane-like Ti-oxo clusters ([Ti_8_O_12_(COO)_12_]). The TiZr-oxo cluster was consisted of a Ti_8_-cube ([Ti_8_O_4_]^24+^) and two Zr-pyramid ([ZrO_4_]^4−^) on the top and bottom. Each of the eight *μ*_3_-O on the top and bottom connected the Zr^4+^ with two Ti^4+^ to form two rectangular pyramid structures, while each of the four *μ*_2_-O in the middle bridged two Ti^4+^ to make them as a whole. Thus, compared with Zr-oxo clusters, the TiZr-oxo clusters could be obtained through replacing the Zr_4_-square by the Ti_8_-cube. Furthermore, the four carboxylate ligands within the equatorial plane were replaced by eight perpendicular carboxylate ligands [19].

**Fig. 2:**
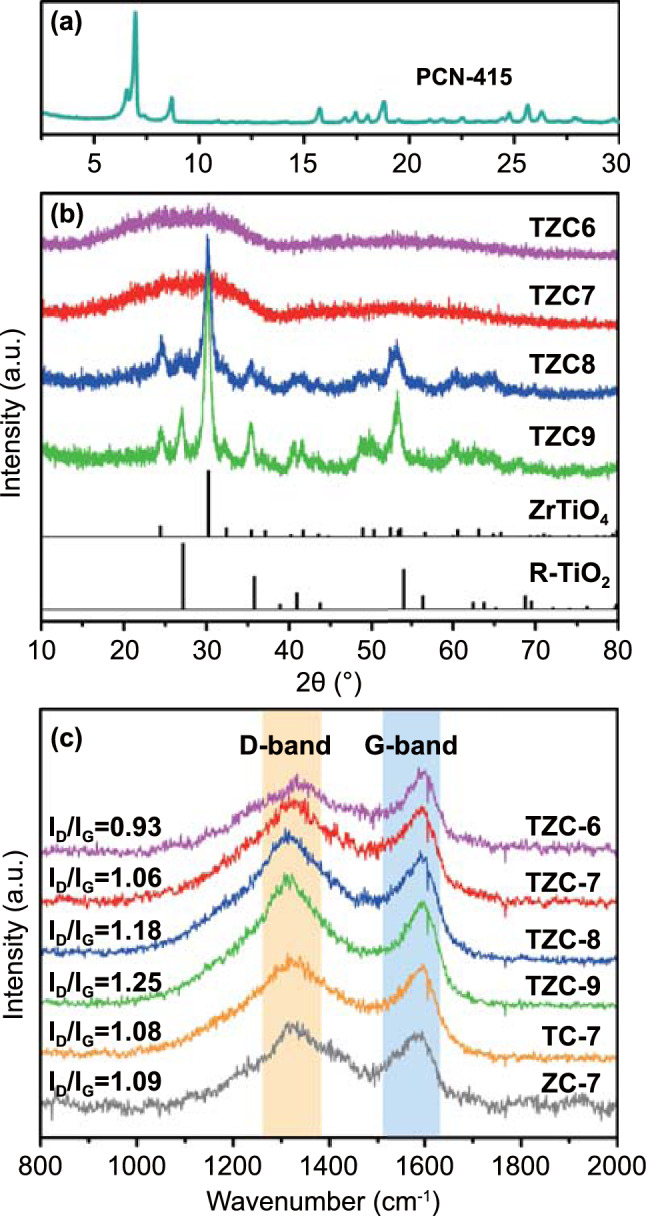
**a** PXRD patterns of PCN-415 and **b** PCN-415 derivatives. **c** Raman spectra of all MOF derivatives

The PXRD patterns shown in Fig. 2a were well in accordance with the Bragg positions from simulative calculation [19], confirming the preparation of pure PCN-415 MOF precursor. The PXRD patterns of TZC-6, TZC-7, TZC-8, and TZC-9 are delineated in Fig. 2b, and those of TC-7 and ZC-7 are shown in Fig. S1. TC-7 composites derived from MIL-125 were consisted of TiO_2_ and carbon, and the TiO_2_ was composed of anatase (JCPDF No. 21–1272) and rutile (JCPDF No. 21–1276) phases. The mixed TiO_2_ phases resulted from the incomplete transition from anatase completely to rutile in high temperature [20]. There was no EMW absorption performance discrepancy between the two types of TiO_2_ [21]. And the experimental evidences and details are shown in Figs. S2 and S3. ZC-7 composites derived from UIO-66 were consisted of tetragonal ZrO_2_ (JCPDS No. 50–1089) and carbon. However, the derivatives of PCN-415 were not a simple combination of TC and ZC. For TZC-8 and TZC-9, the obvious peaks at 24.6° and 30.4° could be identified as the (011) and (111) lattice planes of orthorhombic ZrTiO_4_ (JCPDS No. 34–0415). According to the patterns, all the zirconium ions existed in the form of ZrTiO_4_. The redundant titanium ions transformed into the rutile TiO_2_. However, for TZC-6 and TZC-7, only one wide peak could be identified at round 20–35° in the PXRD patterns, corresponding to the amorphous carbon. The peaks of TiO_2_ and ZrTiO_4_ could not be distinguished, which may be due to the pretty low crystallinity.

In the TGA curves under air atmosphere (Fig. S4), the initial decomposition temperature of TZC composites was higher than TC-7 and ZC-7, demonstrating the superior thermostability of the TZC composites. Neglecting the absorbed water, the carbon contents of TC-7 and ZC-7 were 22.3% and 25.4%, respectively. And the carbon contents of TZC-6, TZC-7, TZC-8, and TZC-9 were 21.2%, 17.1%, 14.9%, and 9.2%, respectively. The reduced carbon content resulted from more carbon escaping from the carbon base at higher temperatures in forms of small molecules. The TiO_2_ contents for TZC-6, TZC-7, TZC-8, and TZC-9 were 42.7%, 44.9%, 46.1%, and 49.2%, while the ZrTiO_4_ contents were 36.1%, 38.0%, 39.0%, and 41.6%, respectively. The detailed calculation equation and analysis were supplied in the supplementary material. Besides, the EDS was also applied to estimate the component contents, which were in good agreement with the above results (Table S1).

For carbon-based materials derived from organic ingredients, the graphitization degree was an essential factor to affect the carbon properties. According to Ferrari and Robertson’s theory [22], the D band was originated from the active *A*_1g_ mode of carbon crystallite boundaries, reflecting the defects and disorders. The G band was attributed to the active *E*_2g_ mode of the infinite crystal, corresponding to the perfect graphite lattices [23, 24]. As the Raman spectrum shown in Fig. 2c, the *I*_D_/*I*_G_ values for TZC-6, TZC-7, TZC-8, and TZC-9 were 0.93, 1.06, 1.18, and 1.25, respectively. This indicated the higher carbonization temperature promoted the formation of small carbon crystallites, enhancing graphitization degrees. And this phenomenon could be observed in many carbon-based composites, especially for MOF derivative [9, 25, 26]. The *I*_D_/*I*_G_ values for TC-7 and ZC-7 were 1.08 and 1.09, similar to TZC-7, illuminating similar graphitization degrees under same carbonization conditions. Generally, the enhanced graphitization degree could directly improve the conductivity, leading to a larger permittivity. Though the larger permittivity would enhance the electrical loss capacity, an over-high conductivity would also result in impedance mismatch to reflect EMWs [16, 27]. Thus, the graphitization degree should be kept within a rational range.

**Fig. 3:**
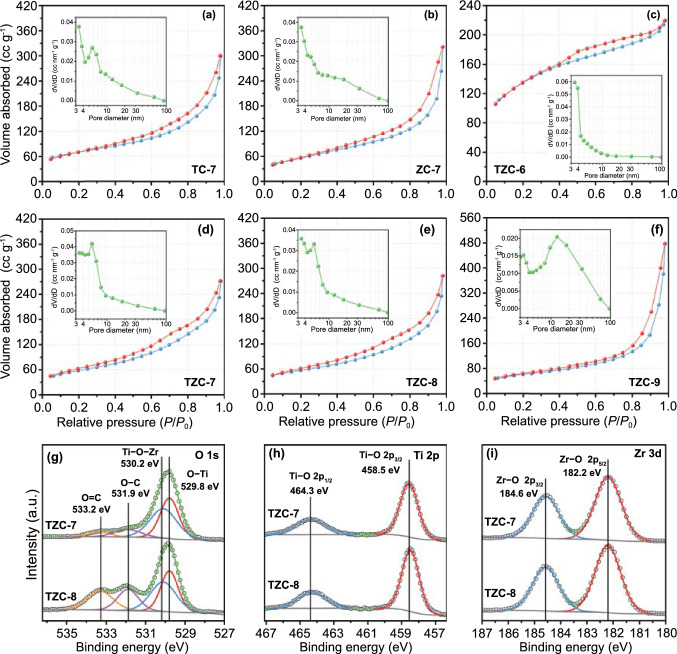
N_2_ absorption (red)–desorption (blue) isotherms with corresponding pore-size distributions in the inset for **a** TC-7, **b** ZC-7, **c** TZC-6, **d** TZC-7, **e** TZC-8, and **f** TZC-9. XPS spectra of TZC-7 and TZC-8: **g** O 1 s spectrum, **h** Ti 2p spectrum, and **i** Zr 3d spectrum

Benefited by the high porosity from framework structures, porous carbon was expected to obtained by in situ carbonization process. The porosity and surface area were determined from the N_2_ absorption–desorption isotherms (Fig. 3a–f). The specific surface areas of TZC-6, TZC-7, TZC-8, and TZC-9 were 483.1, 207.8, 201.5, and 218.7 m^2^ g^−1^, respectively, and those of TC-7 and ZC-7 were 171.6 and 205.5 m^2^ g^−1^. The dominant pore sizes of TZC-6, TZC-7, TZC-8, and TZC-9 were ~ 3, ~ 5, ~ 6, and ~ 12 nm, respectively, increasing with the carbonization temperature. Those of TC-7 and ZC-7 were ~ 6 and ~ 3 nm, respectively. The air inside the pores would reduce the effective permittivity according to the Maxwell–Garnett theories [28].

Due to the absence of characteristic peaks for TZC-7 in PXRD patterns, XPS was applied for further detection. TZC-7 and TZC-8 exhibited perfectly identical survey spectrum with each other (Fig. S5). In O 1 s spectrum (Fig. 3g), the two deconvolution peaks at 529.8 and 530.2 eV were recognized as Ti−O and Ti−O−Zr species, reflecting the existence of TiO_2_ and ZrTiO_4_, respectively [29]. The two deconvolution peaks at 531.9 and 533.2 eV represented O−C and O=C bonds, respectively [17, 30, 31], originating from the small number of functional groups such as oxhydryl and carbonyl in carbon base, which could also be elaborated by the FT-IR spectra (Fig. S6). In Fig. 3h, the signals located at 458.5 and 464.3 eV were identified as Ti 2p_3/2_ and Ti 2p_1/2_ species, respectively, confirming the Ti−O bonds [32, 33]. In Fig. 3i, the signals located at 182.2 and 184.6 eV were recognized as Zr 2p_5/2_ and Zr 2p_3/2_ species, respectively, corresponding to the Zr−O bonds [16, 29]. Thus, we could confirm the component categories of TZC-7 were constituted by carbon, TiO_2_, and ZrTiO_4_, the same as TZC-8.

**Fig. 4:**
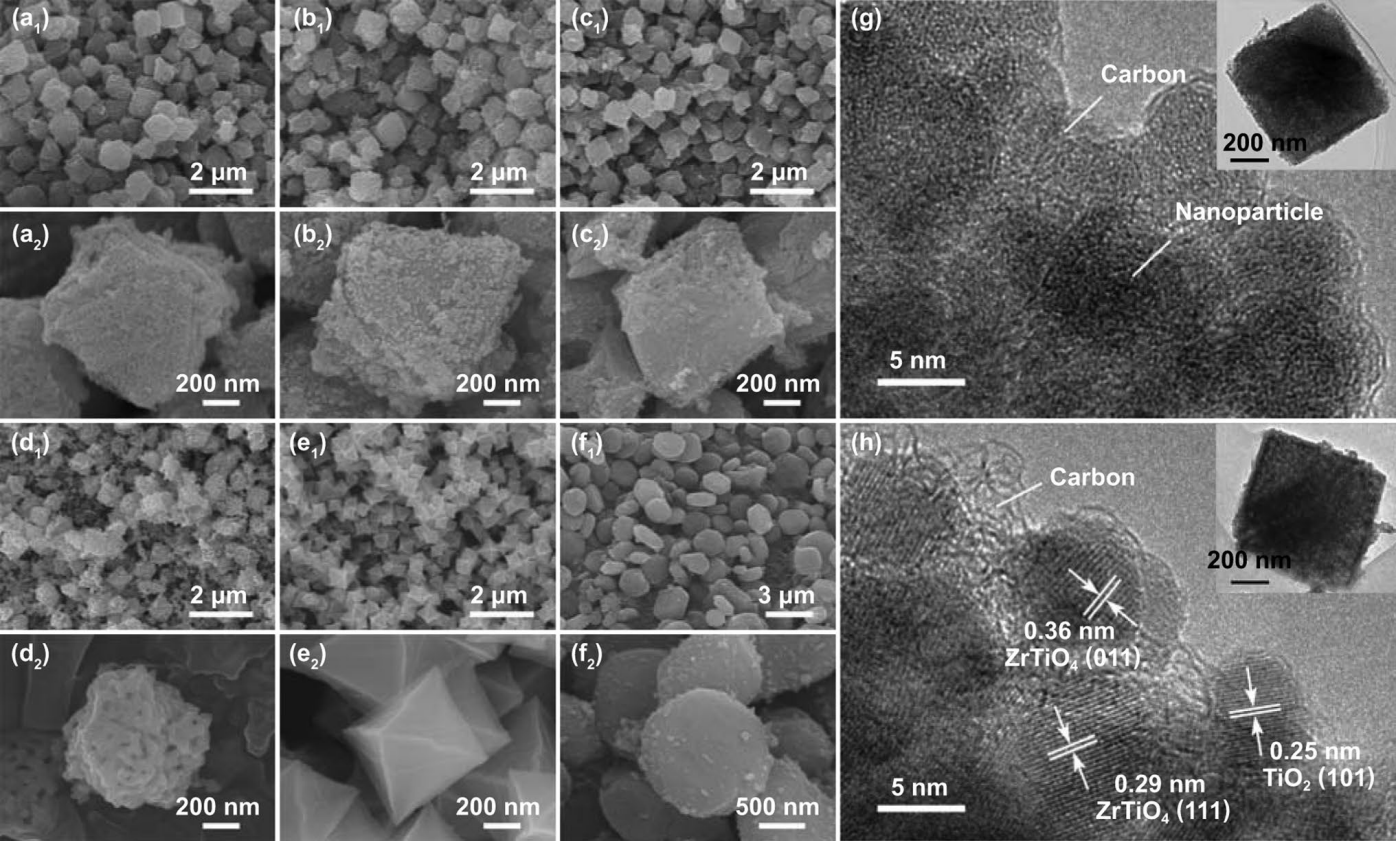
SEM images of **a** TZC-6, **b** TZC-7, **c** TZC-8, **d** TZC-9, **e** ZC-7, and **f** TC-7. HR-TEM images of **g** TZC-7, and **h** TZC-8

The micro-morphologies of all carbon-based composites were characterized by FE-SEM and depicted in Fig. 4. All TZC composites showed up octahedral microstructures with an average edge length of 1 μm. The surface appearance and contour of TZC-9 were different from the other three due to the reduced carbon content and strongly sintered oxide nanoparticles. ZC-7 was also octahedral, but the average edge length was only 600 nm. Besides, it possessed a smooth and sunken surface because the higher crystallinity of ZrO_2_ than ZrTiO_4_ would increase the compactness and reduce the pore sizes. Therefore, even with similar structures, TZC composites would be easier to achieve the impedance matching. Incidentally, TC-7 sample exhibited much larger pie-like particles with an average diameter of 1.5 μm.

Taking TZC-7 and TZC-8 as examples, the interior structures are delineated in Fig. 4g, h. The ZrTiO_4_ and TiO_2_ nanoparticles with a diameter of 5–10 nm were uniformly embedded inside the solid octahedral particles. Amorphous carbon was coated around the nanoparticles. The crystal lattices were ambiguous for TZC-7, reconfirming the pretty low crystallinity of ZrTiO_4_ and TiO_2_. For TZC-8, the interplanar spacings of 0.29 and 0.36 nm were ascribed to the (111) and (011) lattice planes of ZrTiO_4_; the interplanar spacings of 0.25 nm were assigned to the (101) lattice planes of TiO_2_. Numerous nanoparticles with carbon base forming huge contact areas would signally promote the interfacial polarization to enhance attenuation capacity.

### Electromagnetic Performance and Parameter

**Fig. 5:**
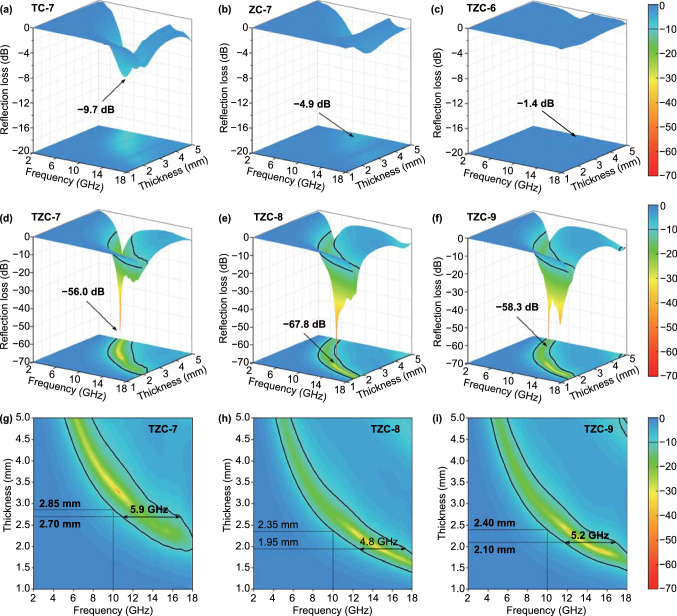
Three-dimensional RL representations of **a** TC-7, **b** ZC-7, **c** TZC-6, **d** TZC-7, **e** TZC-8, and **f** TZC-9. Two-dimensional RL projection mappings of **g** TZC-7, **h** TZC-8, and **i** TZC-9

The EMW absorption performances were evaluated by RL values, and calculated based on the transmission line theory in the metal backboard model by Eqs. (1) and (2) [13, 34, 35]:

1 $$Z_{in} = Z_{0} \sqrt {\frac{{\mu_{r} }}{{\varepsilon_{r} }}} \tanh \left( {\frac{2\pi jfd}{c}\sqrt {\mu_{r} \varepsilon_{r} } } \right)$$

2 $$RL = 20\log \left| {\frac{{Z_{in} - Z_{0} }}{{Z_{in} + Z_{0} }}} \right|$$

in which *Z*_in_ and *Z*_0_ represented the input impedance and free space impedance; *ε*_r_ and *μ*_r_ referred to the relative complex permittivity and permeability; *c* and were the vacuous light velocity and absorber thickness, respectively. The three-dimensional RL representations (Fig. 5) intuitively revealed the minimum *RL* values for TC-7 and ZC-7 were − 9.7 dB at 4.4 mm and*d* − 1.2 dB at 4.8 mm, signifying poor absorption rates and large thickness. Some reports proved these MOF derivatives could exhibit a better absorption intensity, but higher filling rates were required [16, 36], meaning a larger material density. The unsatisfactory *RL* values for TZC-6 were − 1.4 dB (4.8 mm, 10.3 GHz), resulting from the poor conductivity of low-temperature-treated carbon [27]. However, the *RL* values for TZC-7, TZC-8, and TZC-9 were − 56.0 dB (3.28 mm, 10.5 GHz), − 67.8 dB (2.16 mm, 13.0 GHz), and − 58.3 dB (2.37 mm, 12.0 GHz), respectively, attesting the huge potential in absorption intensity. The maximum EAB for TZC-7, TZC-8, and TZC-9 was 5.9 GHz at 2.70 mm, 4.8 GHz at 1.95 mm, and 5.2 GHz at 2.10 mm, respectively. Thus, TZC-7 exhibited the optimum bandwidth performance, which was superior than many reported EMW absorption materials as well. A reasonable thickness comparison required a fixed frequency, as the thickness should reduce inevitably with the increasing frequency. The minimum thickness to guarantee RL values less than − 10 dB at 10.0 GHz was 2.85 mm for TZC-7, 2.35 mm for TZC-8, and 2.40 mm for TZC-9, respectively. The smaller matching thickness of TZC-8 was attributed to stronger loss capacities and more optimized impedance matching characteristics.

**Fig. 6:**
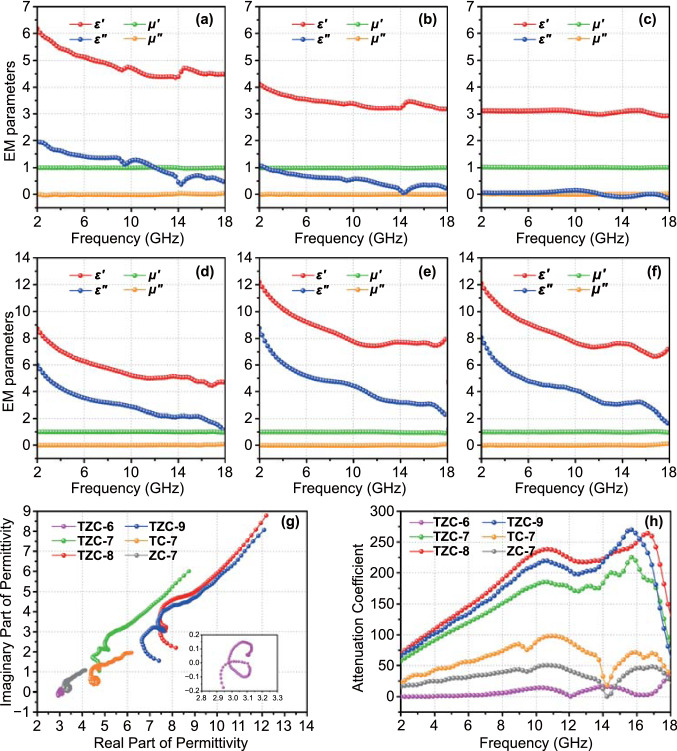
Electromagnetic parameters of **a** TC-7, **b** ZC-7, **c** TZC-6, **d** TZC-7, **e** TZC-8, and **f** TZC-9. **g** Cole–Cole plots, and **h** attenuation coefficient of all MOF derivatives

To expose the inner mechanisms of the excellent absorption performances, the crucial electromagnetic parameters (*ε*_r_ = *ε′* − *jε″*, *μ*_r_ = *μ'* − *jμ''*) were measured out and shown in Fig. 6a–f. In general, the real parts and imaginary parts of electromagnetic parameters were on behalf of the EMW energy shortage and dissipation, respectively [17, 37, 38]. For all composites, the real parts (*μ'*) and the imaginary parts (*μ''*) of permeability kept 1 and 0 over the whole frequency range measured, reflecting their non-magnetic natures. The real parts of permittivity (*ε'*) of TC-7 and ZC-7 decreased from 6.2 to 4.5, and 4.1 to 3.2, while the imaginary parts (*ε''*) decreased from 1.9 to 0.5, 1.0 to 0.2, respectively. Especially for TZC-6, the *ε'* and *ε''* values were below 3.2 and 0.3, respectively, due to the pretty small conductivity, which corresponded to the low graphitization degree in Raman analysis. Though the low permittivity was usually regarded as the prerequisites for EMWs entering absorbers, it would lead to a weak attenuation ability as well. And the latter was the primary reason for the weak absorption intensity for the three studied samples. Nevertheless, the permittivity of TZC-7, TZC-8, and TZC-9 achieved many enhancements. Precisely, the *ε'* values declined from 8.7 to 4.5 for TZC-7, 12.2 to 8.1 for TZC-8, and 12.1 to 7.4 for TZC-9. In the meantime, the *ε''* values declined from 6.0 to 1.0 for TZC-7, 8.8 to 2.2 for TZC-8, and 8.1 to 1.6 for TZC-9. Thus, the dielectric storage and attenuation capabilities of TZC-7, TZC-8, and TZC-9 were signally enhanced. The enhancements were mainly attributed to the increased graphitization degrees. Besides, TZC-8 possessed the largest imaginary part, which guaranteed its optimal absorption intensity and matching thickness. Though TZC-9 exhibited the largest graphitization degree, no improvement in permittivity was achieved due to the reduction of carbon content.

### EMW Absorption Mechanism

Generally, the polarization loss (*ε*_p_*''*) and conductive loss (*ε*_c_*''*) were regarded as the two essentials in dielectric dissipation. And the *ε''* was usually expressed as Eq. (3) [34, 39, 40]:

3 $$\varepsilon ^{\prime\prime}{ = }\varepsilon_{p} ^{\prime\prime} + \varepsilon_{c} ^{\prime\prime} = \omega \tau \frac{{\varepsilon_{s} - \varepsilon_{\infty } }}{{1 + \omega^{2} \tau^{2} }} + \frac{\sigma }{{\varepsilon_{0} \omega }}$$

where *ω*, *τ*, and *σ* referred to the angular frequency, polarization relaxation time, and conductivity; *ε*_0_ represented the permittivity of vacuum; *ε*_s_ and *ε*_∞_ stood for the permittivity at electrostatic field and high-frequency limit, respectively. Considering the inverse relation between conductivity and frequency, the overall downward trends in *ε''* curves pointed out the conductivity were conspicuous and the conductive loss actually made sense in electromagnetic energy attenuation. The conductivity came from the electronic migration in graphite plane and the electron transition between nanocrystalline graphite [41]. For all TZC composites, two broad resonance peaks at around 9.5 and 15.5 GHz could also be observed, which were usually considered to be connected with the polarizations. Due to the nonnegligible conductivity, the original Debye polarization–relaxation model was not suitable any more. Therefore, combined with above equation about *ε"* value, the semicircle equation should be modified as Eq. (4) [17, 21]:

4 $$\left( {\varepsilon^{\prime} - \frac{{\varepsilon_{s} - \varepsilon_{\infty } }}{2}} \right)^{2} + \left( {\varepsilon ^{\prime\prime} - \frac{\sigma }{{\varepsilon_{0} \omega }}} \right)^{2} = \left( {\frac{{\varepsilon_{s} - \varepsilon_{\infty } }}{2}} \right)^{2}$$

Thus, in the *ε'' vs. ε'* curves (Cole–Cole plots), the standard downward semicircles to represent the polarization relaxation process would be distorted, and the end of the curves would extend to the upper right like a “tail” [42]. All TZC composites exhibited two obvious semicircles while TC-7 and ZC-7 only plotted one (Fig. 6g), which illuminated TZC composites were qualified with more polarization–relaxation processes. These processes originated from the more electronegativity differences between heterogeneous particles and carbon bases to induce more interface polarization process. To verify this point, *CST Microwave Studio* was applied to simulate the electromagnetic behaviors on a particle. The animation (Suppporting Information) records the changed surface current intensity and indicated that the surface current intensity on the ZrTiO_4_/carbon and TiO_2_/carbon interfaces got much enhanced under the excitation of alternating electric fields. And the more evident effect on the ZrTiO_4_/carbon surfaces signified a stronger interfacial polarization behavior as shown in the captured images (Fig. S7). Besides, the microstructure of fine nanoparticles dispersing in carbon base would strongly enlarge the polarization effect to strengthen the electric field attenuation. The “tail” gradient on the *ε'' vs. ε'* curves of TZC-7 was much larger than these of TC-7 and ZC-7, clarifying the conductive loss superiority of TZC composites. Additionally, the absence of the tail for TZC-6 and the smaller slope of TZC-7 than TZC-8 reflected the conductivity discrepancies of carbon bases, following the rule that high carbonization temperature promoted the graphitization to enhance conductivity [43, 44]. The measured conductive properties (Table S2) implied that TZC-8 and TZC-9 possessed higher conductive loss than TZC-7, TC-7, and ZC-7. And the conductive loss in TZC-6 was negligible. These results were well accordant with the gradient on the ε'' vs. ε' curves as well. Furthermore, the attenuation coefficient (*α*) was calculated according to Eq. (5) to compare the overall attenuation capacity (Fig. 6h) [45].

5 $$\alpha = \frac{\sqrt 2 \pi f}{c} \times \sqrt {\left( {\mu^{\prime\prime}\varepsilon^{\prime\prime} - \mu ^{\prime}\varepsilon ^{\prime}} \right) + \sqrt {\left( {\mu^{\prime\prime}\varepsilon^{\prime\prime} - \mu ^{\prime}\varepsilon ^{\prime}} \right)^{2} + \left( {\mu^{\prime\prime}\varepsilon^{\prime} - \mu ^{\prime}\varepsilon ^{\prime\prime}} \right)^{2} } }$$

TZC-7, TZC-8, and TZC-9 possessed much higher *α* values than the other three composites, confirming their strong electromagnetic energy attenuation abilities again. With all dielectric behaviors being analyzed, we drawn the conclusion that the excellent attenuation performances of TZC composites benefited from the synergies of the enhanced polarization losses and strong conductive loss, and this fundamentally stemmed from the specific microstructures and rational component collocation.

**Fig. 7:**
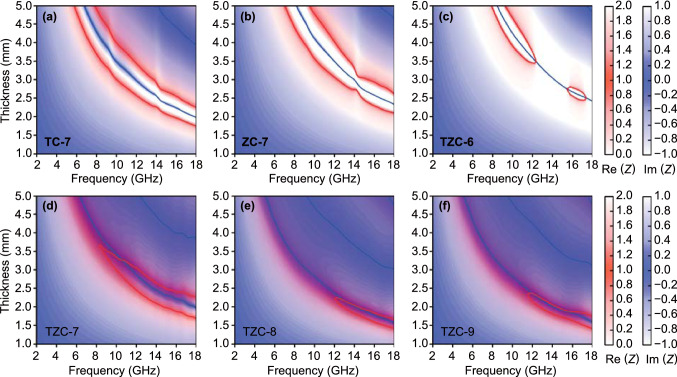
Two-dimensional projection drawings of (*Re*(*Z*)) and (*Im*(*Z*)) values *versus* thickness and frequency for **a** TC-7, **b** ZC-7, **c** TZC-6, **d** TZC-7, **e** TZC-8, and **f** TZC-9

Besides the attenuation capacity, the impedance matching characteristic was the other crucially important factor to determine the EMW absorption performance, because it directly affected the EMW behaviors on the incident interface. To ensure all incident waves entering inside the absorbers and not being reflected, a perfect impedance matching condition commands the input impedance (*Z*_in_) is equal to free space impedance (*Z*_0_) [3]. To compare the discrepancies between *Z*_in_ and *Z*_0_, *Z* values (*Z* = *Z*_in_ / *Z*_0_) were calculated, and the two-dimensional projection mappings of the real parts (*Re*(*Z*)) and imaginary parts (*Im*(*Z*)) for all composites were delineated in Fig. 7. In this case, a good impedance matching required the *Re*(*Z*) value and *Im*(*Z*) value was equal to 1 and 0, respectively (purple areas on the mappings). For TC-7, ZC-7, and TZC-6, no purple area could be observed, indicating an impedance mismatching. However, it did not fit the general perception that materials with low permittivity possessed better impedance characters. The key reason was the weak attenuation abilities. In this situation, though most EMWs could enter into absorbers, it would not be effectively consumed. After the reflection on absorber–metal interface, the EMWs passed through the front surface again. TZC-7, TZC-8, and TZC-9 possessed the more optimized impedance matching characteristic. The purple areas of TZC-8 and TZC-9 located on the thin-thickness regions than TZC-7, but their narrow regions also resulted from the imperfect EAB performances than TZC-7. The maximum of (| *Z*_in_/*Z*_0_ |) values at 3.0 mm (Fig. S8) for TZC-8 and TZC-9 could not reach 1.0 but that for TZC-7 was much closer to 1.0 within certain frequency range, which also indicated TZC-7 was qualified with a better impedance matching characteristic.

The quarter-wavelength model (Eq. (6) could be used to explain the EMW absorption performances [11, 46, 47].

6 $$t_{TM} = \frac{nc}{{4f\sqrt {\left| {\varepsilon_{r} } \right|\left| {\mu_{r} } \right|} }} n = 1, 3, 5 \ldots$$

The theoretical matching thickness (*t*_TM_) curves for TZC-7, TZC-8, and TZC-9 were drawn on the two-dimensional *RL* projection mappings (Fig. S9). The *t*_TM_ curves were well accordance with the strong absorption regions, indicating the EMW absorption behavior followed the quarter-wavelength law [48]. The description of absorption behaviors from the view of the EMWs was as followed. When EMWs were incident into the air–absorber interface, most EMWs enter into the absorber and a small amount were reflected. During the process that EMWs are totally reflected by absorber–metal interface and subsequently come back to the front surface, the EMWs are consumed to make the amplitude reduced. If these EMWs in the absorber and the reflected waves in the air are equipped with equal amplitude and opposite phase on the air–absorber interface, the destructive interference will lead to the disappearance of reflected waves and enhance the waves which are back into the absorber.

**Fig. 8:**
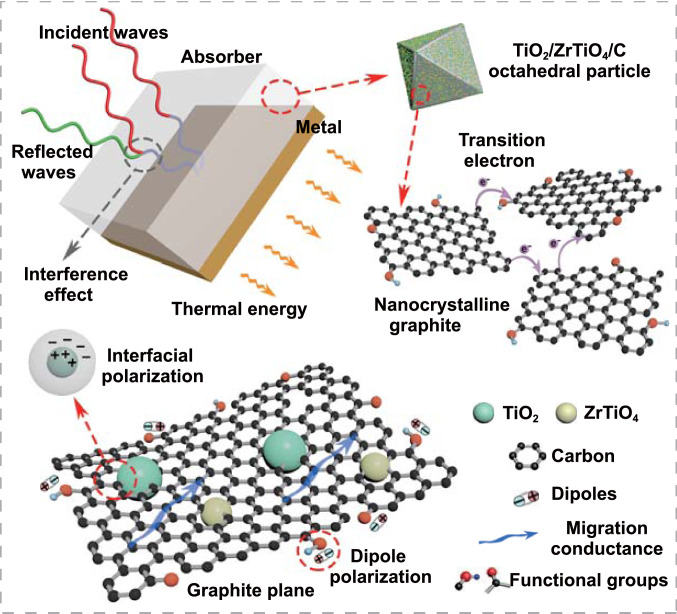
Schematic diagram of the EMW absorption behaviors and mechanisms for the TiO_2_/ZrTiO_4_/carbon composites

As a summary of all above analysis, the EMW absorption behaviors and mechanisms of TZC composites are delineated in Fig. 8 and stated as followed. Firstly, the carbon base provided essential conductivity. Its conductive attenuation capacity could be directly affected by graphitization degrees, and finally regulated by carbonization temperatures. Secondly, the TiO_2_ and ZrTiO_4_ were introduced to optimize the inherent impedance mismatching of carbon, and their uniformly distributed nanoparticles provided abundant phase interface to enhance the interfacial polarization loss. Thirdly, the matching thickness and absorption bandwidth were determined by impedance matching characteristic, which could also be adjusted by carbonization temperatures. Additionally, the dipole polarization induced by functional groups, and the impedance adjustment arising from porosity would contribute to the EMW absorption performances as well.

## Conclusions

Porous carbon-wrapped TiO_2_/ZrTiO_4_ composites derived from bimetallic MOFs (PCN-415) were successfully prepared for efficient EMW absorption. The ZrTiO_4_ and TiO_2_ nanoparticles with the diameter of 5–10 nm were uniformly embedded inside the solid amorphous carbon octahedral particles. The EMW absorption performances could be regulated by carbonization temperature. The composites treated at 800 °C were qualified with a strong absorption intensity of − 67.8 dB (2.16 mm, 13.0 GHz), and those treated at 700 °C possessed a wide EAB of 5.9 GHz (2.70 mm). The essential conductive loss from carbon base and the enhanced interfacial polarization from TiO_2_ and ZrTiO_4_ nanoparticles achieved the synergy among compositions, structures, and functions, which ensured the satisfactory performances. This work exhibited performance advantages of PCN-415 derived TiO_2_/ZrTiO_4_/carbon composites and certified a huge potential of non-magnetic bimetallic MOF derivatives in EMW absorption applications.

## Supplementary information

Supplementary file1 (PDF 1043kb)

## References

[1] Chen H, Ma W, Huang Z, Zhang Y, Huang Y (2019). Graphene-based materials toward microwave and terahertz absorbing stealth technologies. Adv. Opt. Mater..

[2] Li Q, Zhang Z, Qi L, Liao Q, Kang Z (2019). Toward the application of high frequency electromagnetic wave absorption by carbon nanostructures. Adv. Sci..

[3] Li Y, Liu X, Nie X, Yang W, Wang Y (2019). Multifunctional organic-inorganic hybrid aerogel for self-cleaning, heat-insulating, and highly efficient microwave absorbing material. Adv. Funct. Mater..

[4] Zhang Y, Huang Y, Zhang T, Chang H, Xiao P (2015). Broadband and tunable high-performance microwave absorption of an ultralight and highly compressible graphene foam. Adv. Mater..

[5] Koo WT, Yu S, Choi SJ, Jang JS, Cheong JY (2017). Nanoscale PdO catalyst functionalized Co_3_O_4_ hollow nanocages using MOF templates for selective detection of acetone molecules in exhaled breath. ACS Appl. Mater. Interfaces.

[6] Liao Q, He M, Zhou Y, Nie S, Wang Y (2018). Highly cuboid-shaped heterobimetallic metal-organic frameworks derived from porous Co/Zno/C microrods with improved electromagnetic wave absorption capabilities. ACS Appl. Mater. Interfaces.

[7] Dang S, Zhu Q-L, Xu Q (2017). Nanomaterials derived from metal-organic frameworks. Nat. Rev. Mater..

[8] Wang K, Chen Y, Tian R, Li H, Zhou Y (2018). Porous Co-C core-shell nanocomposites derived from Co-MOF-74 with enhanced electromagnetic wave absorption performance. ACS Appl. Mater. Interfaces.

[9] Wu N, Xu D, Wang Z, Wang F, Liu J (2019). Achieving superior electromagnetic wave absorbers through the novel metal-organic frameworks derived magnetic porous carbon nanorods. Carbon.

[10] Wu N, Lv H, Liu J, Liu Y, Wang S (2016). Improved electromagnetic wave absorption of Co nanoparticles decorated carbon nanotubes derived from synergistic magnetic and dielectric losses. Phys. Chem. Chem. Phys..

[11] Li X, Feng J, Du Y, Bai J, Fan H (2015). One-pot synthesis of CoFe_2_O_4_/graphene oxide hybrids and their conversion into FeCo/graphene hybrids for lightweight and highly efficient microwave absorber. J. Mater. Chem. A.

[12] Liu L, He N, Wu T, Hu P, Tong G (2019). Co/C/Fe/F hierarchical flowers with strawberry-like surface as surface plasmon for enhanced permittivity, permeability, and microwave absorption properties. Chem. Eng. J..

[13] Zhao B, Li Y, Zeng Q, Wang L, Ding J (2020). Galvanic replacement reaction involving core-shell magnetic chains and orientation-tunable microwave absorption properties. Small.

[14] Liu Q, Cao Q, Bi H, Liang C, Yuan K (2016). CoNi@SiO_2_@TiO_2_ and CoNi@air@TiO_2_ microspheres with strong wideband microwave absorption. Adv. Mater..

[15] Wu Q, Jin H, Chen W, Huo S, Chen X (2018). Graphitized nitrogen-doped porous carbon composites derived from ZIF-8 as efficient microwave absorption materials. Mater. Res. Express.

[16] Zhang X, Qiao J, Liu C, Wang F, Jiang Y (2020). A MOF-derived ZrO_2_/C nanocomposite for efficient electromagnetic wave absorption. Inorg. Chem. Front..

[17] Qiao J, Zhang X, Xu D, Kong L, Lv L (2020). Design and synthesis of TiO_2_/Co/carbon nanofibers with tunable and efficient electromagnetic absorption. Chem. Eng. J..

[18] Zhang N, Huang Y, Wang M (2018). Synthesis of graphene/thorns-like polyaniline/alpha-Fe_2_O_3_@SiO_2_ nanocomposites for lightweight and highly efficient electromagnetic wave absorber. J. Colloid Interface Sci..

[19] Yuan S, Qin JS, Xu HQ, Su J, Rossi D (2018). [Ti_8_Zr_2_O_12_(COO)_16_] cluster: an ideal inorganic building unit for photoactive metal-organic frameworks. ACS Cent. Sci..

[20] Secundino-Sánchez O, Diaz-Reyes J, Aguila-López J, Sánchez-Ramírez JF (2019). Crystalline phase transformation of electrospinning TiO_2_ nanofibres carried out by high temperature annealing. J. Mol. Struct..

[21] Qiao J, Zhang X, Liu C, Lyu L, Wang Z (2020). Facile fabrication of Ni embedded TiO_2_/C core-shell ternary nanofibers with multicomponent functional synergy for efficient electromagnetic wave absorption. Compos. B: Eng..

[22] Ferrari AC, Rodil SE, Robertson J (2000). Interpretation of Raman spectra of disordered and amorphous carbon. Phys. Rev. B.

[23] Tuinstra F, Koenig JL (1970). Raman spectrum of graphite. J. Chem. Phys..

[24] Nemanich RJ, Solin SA (1979). First- and second-order Raman scattering from finite-size crystals of graphite. Phys. Rev. B.

[25] Xu X, Ran F, Fan Z, Cheng Z, Lv T (2020). Bimetallic metal-organic framework-derived pomegranate-like nanoclusters coupled with CoNi-doped graphene for strong wideband microwave absorption. ACS Appl. Mater. Interfaces.

[26] Xu H, Yin X, Li M, Ye F, Han M (2018). Mesoporous carbon hollow microspheres with red blood cell like morphology for efficient microwave absorption at elevated temperature. Carbon.

[27] Zhang X, Qiao J, Wang F, Lv L, Xu D (2020). Tailoring electromagnetic absorption performances of TiO_2_/Co/carbon nanofibers through tuning graphitization degrees. Ceram. Int..

[28] Zhao B, Guo X, Zhao W, Deng J, Fan B (2017). Facile synthesis of yolk–shell Ni@void@SnO_2_(Ni_3_Sn_2_) ternary composites via galvanic replacement/Kirkendall effect and their enhanced microwave absorption properties. Nano Res..

[29] Hiroyuki Ikawa TY, Kojima K, Matsumoto S (1991). X-ray photoelectron spectroscopy study of high- and low-temperature forms of zirconium titanate. J. Am. Ceram. Soc..

[30] Yin Y, Liu X, Wei X, Yu R, Shui J (2016). Porous CNTs/Co composite derived from zeolitic imidazolate framework: a lightweight, ultrathin, and highly efficient electromagnetic wave absorber. ACS Appl. Mater. Interfaces.

[31] Ran F, Xu X, Pan D, Liu Y, Bai Y (2020). Ultrathin 2D metal–organic framework nanosheets in situ interpenetrated by functional CNTs for hybrid energy storage device. Nano-Micro Lett..

[32] Chi M, Sun X, Sujan A, Davis Z, Tatarchuk BJ (2019). A quantitative XPS examination of UV induced surface modification of TiO_2_ sorbents for the increased saturation capacity of sulfur heterocycles. Fuel.

[33] Ran F, Wang T, Chen S, Liu Y, Shao L (2020). Constructing expanded ion transport channels in flexible MXene film for pseudocapacitive energy storage. Appl. Surface Sci..

[34] Wang L, Huang M, Yu X, You W, Zhang J (2020). MOF-derived Ni_1−x_Co_x_@carbon with tunable nano-microstructure as lightweight and highly efficient electromagnetic wave absorber. Nano-Micro Lett..

[35] Zhang D, Liu T, Cheng J, Cao Q, Zheng G (2019). Lightweight and high-performance microwave absorber based on 2D WS_2_-RGO heterostructures. Nano-Micro Lett..

[36] Ma J, Liu W, Liang X, Quan B, Cheng Y (2017). Nanoporous TiO_2_/C composites synthesized from directly pyrolysis of a ti-based MOFs MIL-125(Ti) for efficient microwave absorption. J. Alloys Compd..

[37] Wang J, Liu L, Jiao S, Ma K, Lv J (2020). Hierarchical carbon fiber@MXene@MoS_2_ core-sheath synergistic microstructure for tunable and efficient microwave absorption. Adv. Funct. Mater..

[38] Sun K, Dong J, Wang Z, Wang Z, Fan G (2019). Tunable negative permittivity in flexible graphene/PDMS metacomposites. J. Phys. Chem. C.

[39] Ning M, Kuang B, Hou Z, Wang L, Li J (2019). Layer by layer 2D MoS_2_/rGO hybrids: an optimized microwave absorber for high-efficient microwave absorption. Appl. Surface Sci..

[40] Liu J, Cao W-Q, Jin H-B, Yuan J, Zhang D-Q (2015). Enhanced permittivity and multi-region microwave absorption of nanoneedle-like ZnO in the X-band at elevated temperature. J. Mater. Chem. C.

[41] Cao M-S, Song W-L, Hou Z-L, Wen B, Yuan J (2010). The effects of temperature and frequency on the dielectric properties, electromagnetic interference shielding and microwave-absorption of short carbon fiber/silica composites. Carbon.

[42] Liu W, Tan S, Yang Z, Ji G (2018). Hollow graphite spheres embedded in porous amorphous carbon matrices as lightweight and low-frequency microwave absorbing material through modulating dielectric loss. Carbon.

[43] Liu W, Liu L, Ji G, Li D, Zhang Y (2017). Composition design and structural characterization of MOF-derived composites with controllable electromagnetic properties. ACS Sustain. Chem. Eng..

[44] Wu Z, Tian K, Huang T, Hu W, Xie F (2018). Hierarchically porous carbons derived from biomasses with excellent microwave absorption performance. ACS Appl. Mater. Interfaces.

[45] Xiang J, Li J, Zhang X, Ye Q, Xu J (2014). Magnetic carbon nanofibers containing uniformly dispersed Fe/Co/Ni nanoparticles as stable and high-performance electromagnetic wave absorbers. J. Mater. Chem. A.

[46] Shu R, Li W, Wu Y, Zhang J, Zhang G (2019). Nitrogen-doped Co-C/MWCNTs nanocomposites derived from bimetallic metal-organic frameworks for electromagnetic wave absorption in the x-band. Chem. Eng. J..

[47] Ren K, Wang Y, Ye C, Du Z, Bian J (2019). Realizing significant dielectric dispersion of composites based on highly conducting silver-coated glass microspheres for wide-band non-magnetic microwave absorbers. J. Mater. Chem. C.

[48] Ma Z, Cao C-T, Liu Q-F, Wang J-B (2012). A new method to calculate the degree of electromagnetic impedance matching in one-layer microwave absorbers. Chinese Phys. Lett..

