# Supplementary material for: Non-Magnetic Bimetallic MOF-Derived Porous Carbon-Wrapped TiO2/ZrTiO4 Composites for Efficient Electromagnetic Wave Absorption
Source: Nanomicro Lett. 2021 Feb 17;13:75. doi: 10.1007/s40820-021-00606-6 (PMC8187513; doi:10.1007/s40820-021-00606-6)
Supplement: Supplementary file 1 — Supplementary file1 (PDF 1043kb) [file 40820_2021_606_MOESM1_ESM.pdf]

Supplementary Information for

Non-Magnetic Bimetallic MOF-Derived Porous Carbon-Wrapped $TiO_2/ZrTiO_4$ Composites for Efficient Electromagnetic Wave Absorption

Jing Qiao[1], Xue Zhang[1], Chang Liu[1], Longfei Lyu[1], Yunfei Yang[1], Zhou Wang[1], Lili Wu[1], Wei Liu[2], Fenglong Wang[1,]*, Jiurong Liu[1,]*

[1]School of Materials Science and Engineering, Shandong University, Jinan 250061, P. R. China

[2]State Key Laboratory of Crystal Materials, Shandong University, Jinan 250100, P. R. China

*Corresponding authors. E-mail: jrliu@sdu.edu.cn (Jiurong Liu); fenglong.wang@sdu.edu.cn (Fenglong Wang)

S1 Supplementary Animation

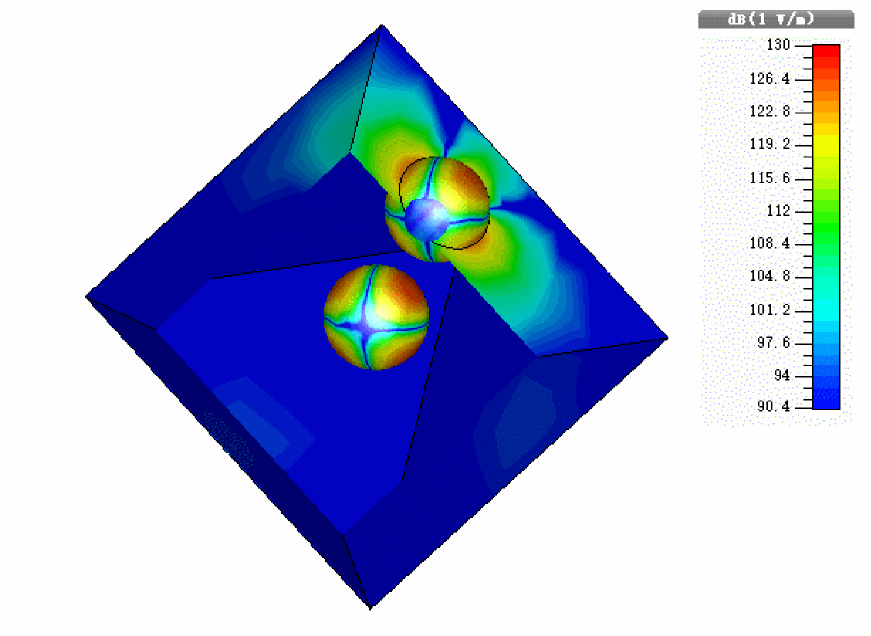

Animation 1 the surface current intensity of simulative $TiO_2/ZrTiO_4$/carbon composites stimulated by electromagnetic waves

CST Microwave Studio is used for discretization and iterative solution of Maxwell equations for a given condition on the basis of time domain finite integration. A simplified model was set with the size and material parameters measured in this work, and the driven frequency is 10 GHz.

S2 Supplementary Tables and Figures

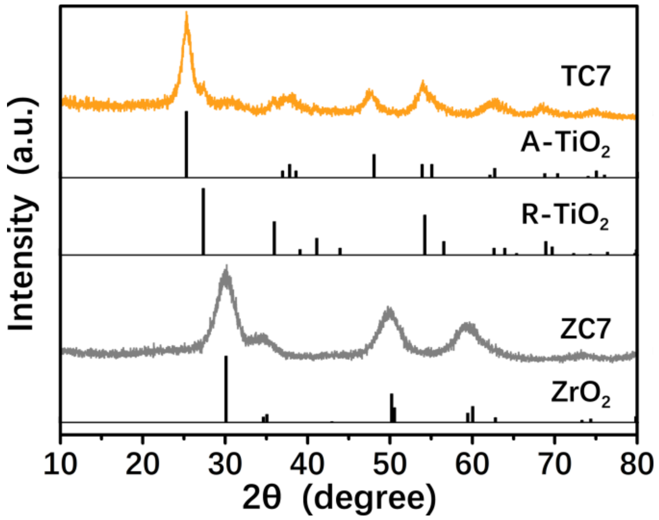

Fig. S1 PXRD patterns of carbon-based MIL-125 and UIO-66 derivatives

Experimental Evidences: To verify the statement that the phase discrepancy of TiO_2 would not affect the absorption performance, the experimental evidences were supplied as followed:

The titanium(IV) isopropoxide was calcined at 600, 700, and 800 °C in air atmosphere. Correspondingly, the XRD patterns (Fig. S2) indicated the anatase phase, mixed phase, and rutile phase TiO_2 were successfully prepared. The electromagnetic parameters of the different TiO_2 (filling rate 50 wt%) were measured (Fig. S3a–c), and the final electromagnetic wave absorption performances were calculated as well (Fig. S3d–f). The permittivity of the three samples was extremely close with each other. And all the samples exhibited similar weak absorption properties.

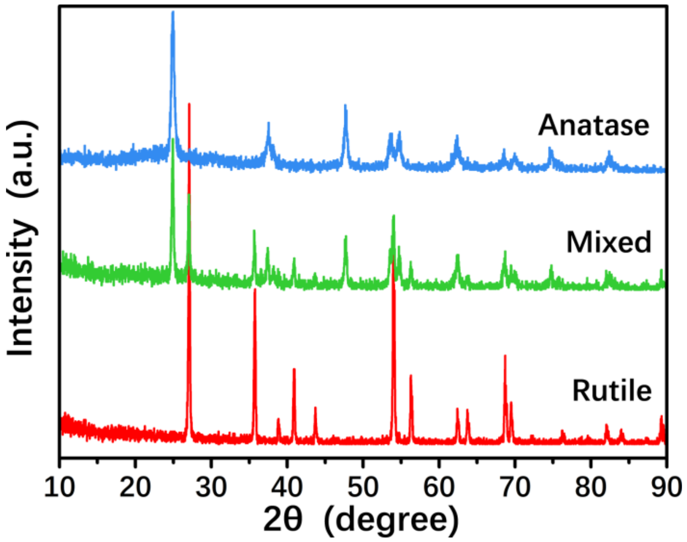

Fig. S2 PXRD patterns of anatase phase, mixed phase, and rutile phase TiO_2

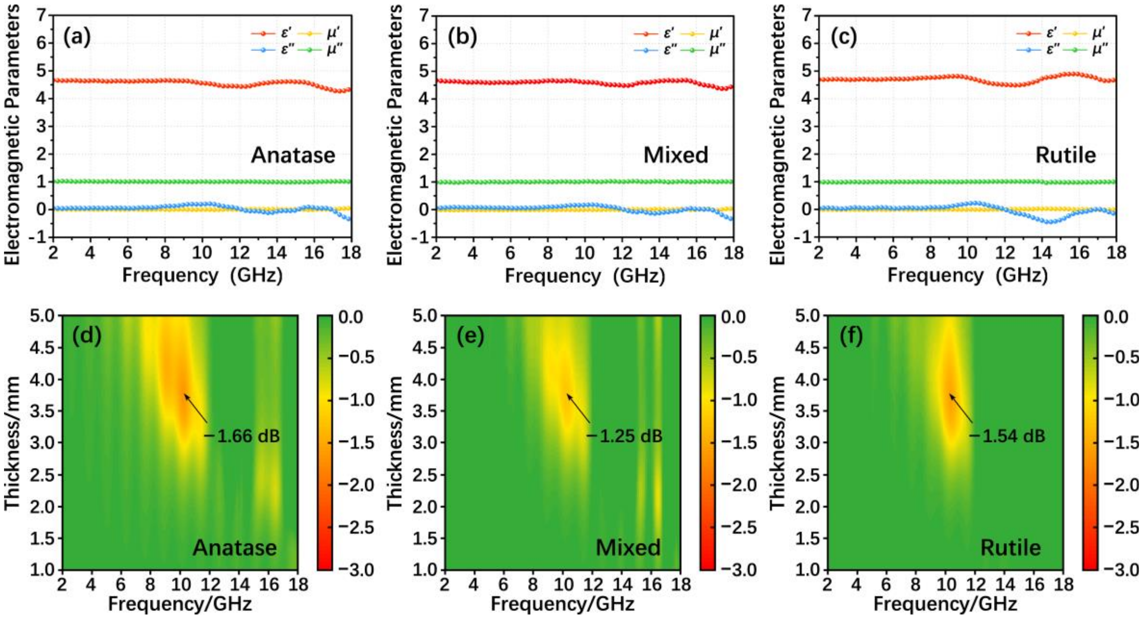

Fig. S3 electromagnetic parameters of **a** anatase phase, **b** mixed phase, and **c** rutile phase TiO_2; and two-dimensional RL projection maps of **d** anatase phase, **e** mixed phase, and **f** rutile phase TiO_2

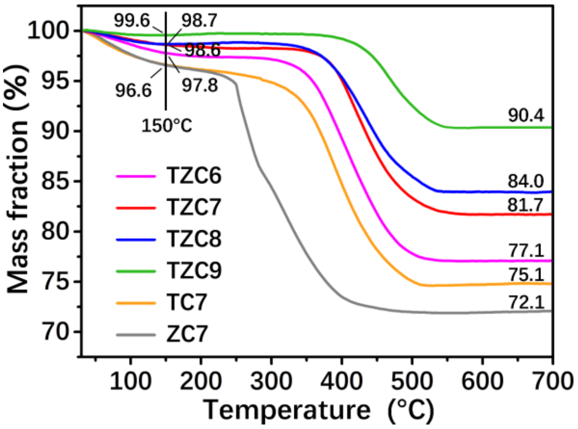

Fig. S4 TGA curves of all MOF derivatives in this work

The detailed calculation equation and analysis of component contents: For the TiO_2/$ZrTiO_4$/carbon composites, the carbon content could be obtained first. In the precursors (PCN-415), the particular MOF crystal structures required the specific TiZr-oxo clusters ($[Ti_8Zr_2O_{12}(COO)_{16}]$). And the atomic ratios between Ti and Zr must be 4:1. Thus, the molar ratio between TiO_2 and $ZrTiO_4$ should be 3:1. Therefore, the weight ratio between TiO_2 and $ZrTiO_4$ should be $3M_{TiO2}$:M_{ZrTiO4}.

The specific calculation equations had been provided as followed. And no mistake could be found after careful checks.

$$wt\%_{\mathrm{TiO_2}} = (1 - wt\%_{\mathrm{Carbon}}) \times \frac{3M_{\mathrm{TiO_2}}}{3M_{\mathrm{TiO_2}} + M_{\mathrm{ZrTiO_4}}}$$

$$wt\%_{\mathrm{ZrTiO_4}} = (1 - wt\%_{\mathrm{Carbon}}) \times \frac{M_{\mathrm{ZrTiO_4}}}{3M_{\mathrm{TiO_2}} + M_{\mathrm{ZrTiO_4}}}$$

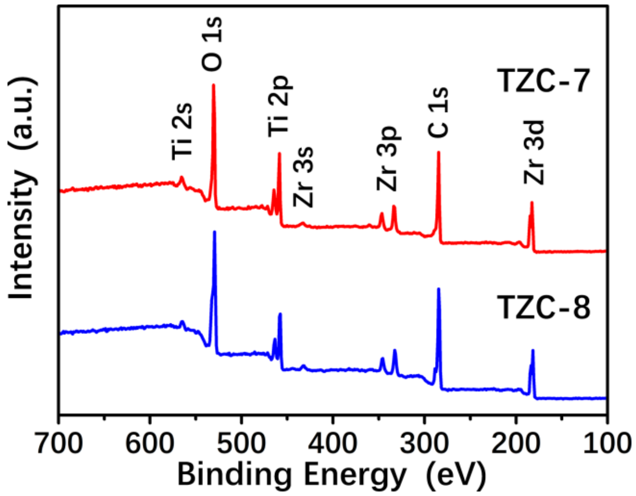

Fig. S5 XPS survey spectrum of TZC-7 and TZC-8

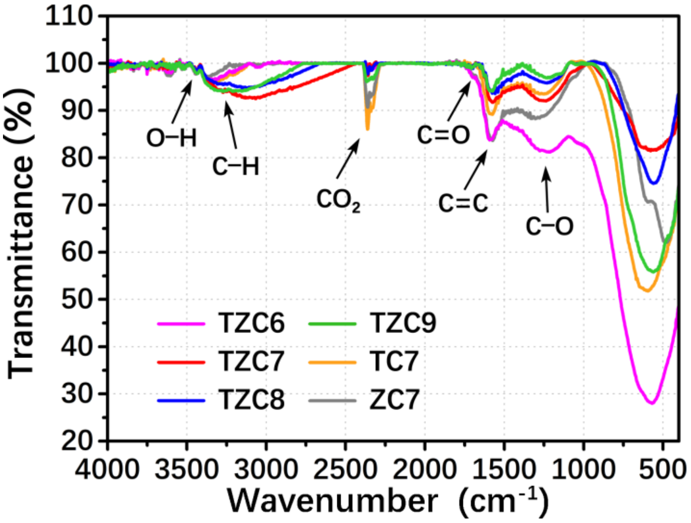

Fig. S6 FT-IR spectra of all MOF derivatives

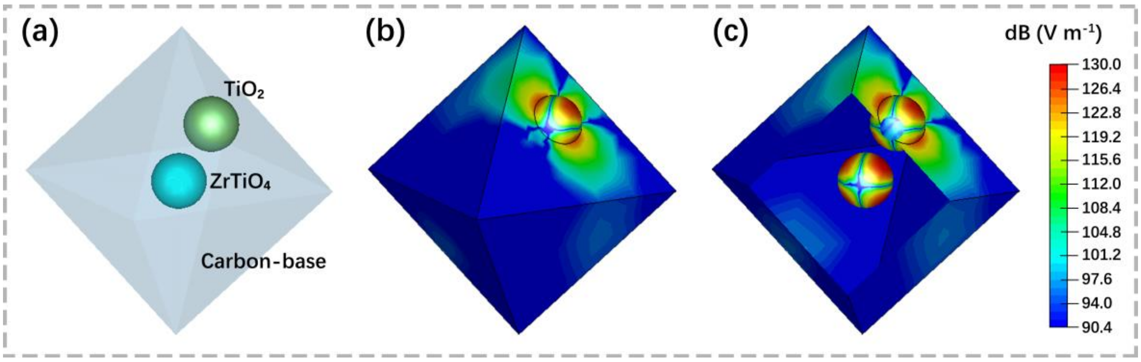

Fig. S7 Simulation results by *CST Microwave Studio*: **a** model structure, **b** surface current intensity, and **c** inner surface current intensity

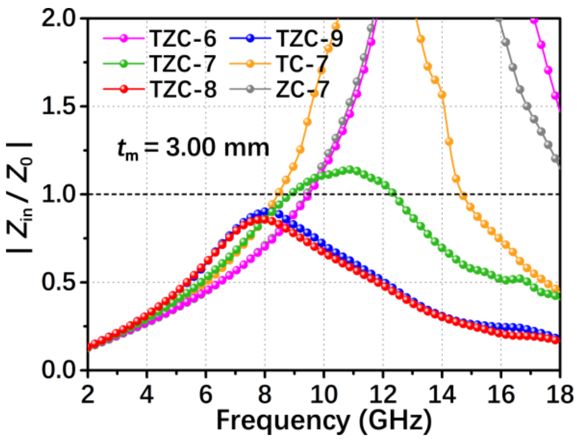

Fig. S8 (| Z_{in} / Z_0 |) curves at 3.00 mm of matching thickness

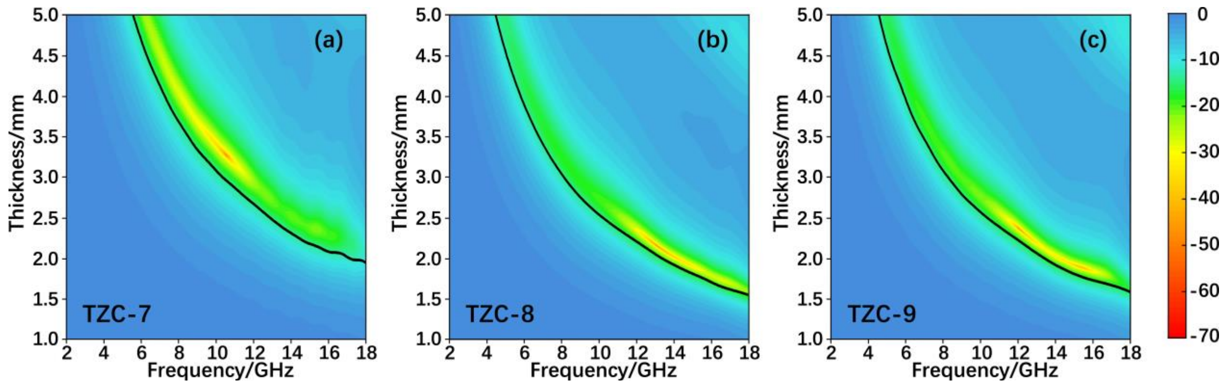

Fig. S9 Theoretical matching thickness curves of **a** TZC-7, **b** TZC-8, and **c** TZC-9 drawn on the two-dimensional RL projection mappings

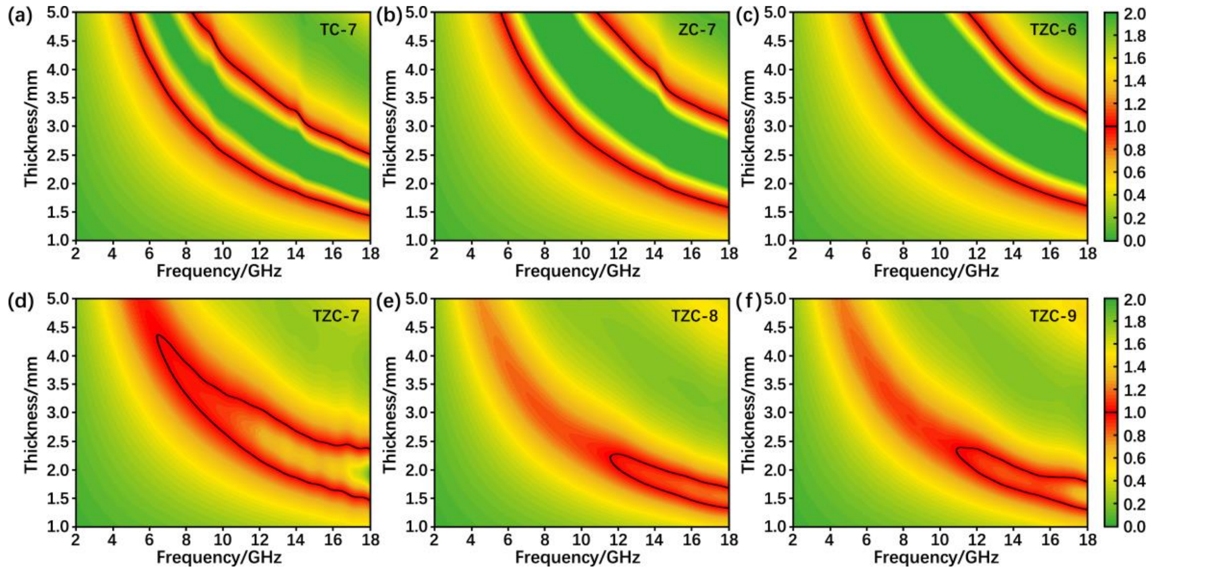

Fig. S10 Two-dimensional (| Z_{in} / Z_0 |) value projection mappings of a **a** TC-7, **b** ZC-7, **c** TZC-6, **d** TZC-7, **e** TZC-8, and **f** TZC-9

Table S1 Atomic molar fractions characterized by EDS, and weight fraction of components

Sample	Atomic molar fraction (%)				Component weight fraction* (wt%)			
	C K	O K	Ti K	Zr L	carbon	TiO_2	$ZrTiO_4$	ZrO_2
TZC-6	38.85	43.11	14.41	3.63	26.60	39.54	33.86	/
TZC-7	34.22	44.90	16.62	4.26	19.94	42.67	37.40	/
TZC-8	25.53	50.19	19.14	5.14	13.34	44.82	41.84	/
TZC-9	16.16	55.86	22.04	5.94	7.17	47.90	44.93	/
TC-7	31.61	49.17	19.22	/	26.42	73.58	/	/
ZC-7	32.27	50.98	/	16.75	24.43	/	/	75.57

* *The component weight fraction was calculated according to the atomic molar fraction.*

Table S2 Conductive properties of all MOF derivatives in this work

Sample	Conductivity (S cm^{-1})	Resistivity (Ω cm)	Sheet Resistance (Ω/□)	Carrier Concentration (cm^{-3})
TZC-6	1.32×10^{-7}	7.60×10^{6}	1.27×10^{11}	1.43×10^{11}
TZC-7	3.70×10^{-1}	2.70×10^{0}	4.51×10^{4}	2.29×10^{19}
TZC-8	7.31×10^{0}	1.37×10^{-1}	2.28×10^{3}	1.41×10^{19}
TZC-9	1.70×10^{0}	5.89×10^{-1}	9.82×10^{3}	2.16×10^{21}
TC-7	5.25×10^{-3}	1.90×10^{2}	3.17×10^{6}	2.36×10^{14}
ZC-7	6.01×10^{-4}	1.66×10^{3}	2.77×10^{7}	4.38×10^{14}
